# Interventions to Improve Antibiotic Use in Hospitals with Different Levels of Complexity in Colombia: Findings from a Before-and-After Study and Suggestions for the Future

**DOI:** 10.3390/antibiotics12050867

**Published:** 2023-05-07

**Authors:** Martha Carolina Valderrama-Rios, Carlos Arturo Álvarez-Moreno, Jorge Alberto Cortes

**Affiliations:** 1 Departamento de Medicina Interna, Facultad de Medicina, Universidad Nacional de Colombia, Bogotá 111321, Colombia; mavalderramar@unal.edu.co (M.C.V.-R.); caalvarezmo@unal.edu.co (C.A.Á.-M.); 2 Clínica Universitaria Colombia, Clínica Colsanitas Grupo Keralty, Bogotá 111321, Colombia; 3 Unidad de Enfermedades Infecciosas, Hospital Universitario Nacional de Colombia, Bogotá 111321, Colombia

**Keywords:** antibacterial drug resistance, antimicrobial stewardship, antibiotic use, strategies, Colombia, lower-middle-income countries

## Abstract

Background: In the collaborative efforts to control bacterial antimicrobial resistance (AMR), the challenge for many low- and middle-income countries currently lies in the adequate design and successful implementation and operation of different strategies aimed at improving antibiotic use during hospital care. This study aims to provide data on these different strategies in three hospitals with different levels of complexity and geographic locations in Colombia. Methods: This before-and-after study describes and analyzes the development and implementation of clinical practice guidelines (CPGs), continuing education courses, quick consultation tools, and antimicrobial stewardship programs (ASPs) with the use of telemedicine. This includes measuring indicators in the ASP framework such as adherence to CPGs and antibiotic consumption. Results: We used five CPGs developed in the Colombian context. We designed and developed a Massive Open Online Course (MOOC) and a mobile application (app) as strategies for dissemination and implementation. The ASP was designed and implemented according to each institution’s level of complexity. In the three hospitals, a progressive increase in adherence to the antibiotic recommendations proposed in the CPGs was observed, and there was a lower use of antibiotics with the ASPs, both in the general wards and ICUs. Conclusions: We concluded that in medium-complexity hospitals located in small rural cities, successful development of ASPs is possible when they are well-planned, implemented, and supported by the organization. It is necessary that Colombia and other Latin American countries continue activities that reduce AMR by designing, implementing, and improving these interventions throughout the national territory.

## 1. Introduction

Bacterial antimicrobial resistance (AMR) development is an important global public health concern [1]. It has been estimated for Latin America and the Caribbean that, in 2019, AMR led to 57.9 deaths per 100,000, with South America as the fourth region with the highest burden of AMR-associated deaths after Sub-Saharan Africa, South Asia, and Eastern Europe [2]. Considering the importance of collaborative efforts between the different sectors involved in antimicrobial use and misuse, the World Health Organization (WHO) published a global action plan on antimicrobial resistance in 2015 [3,4].

Using antimicrobials in hospital environments is a cornerstone for controlling AMR, for which different strategies aimed at improving antibiotic use during hospital care have been proposed. A recommended strategy to start with is to develop clinical practice guidelines (CPGs) for the most common scenarios in daily clinical practice involving antibiotic use, accounting for the national context and local resistance patterns [3,5,6,7]. Another important strategy is to continuously educate the healthcare personnel involved in the prescription, administration, and/or antibiotic control processes on topics related to prudent antibiotic use (e.g., adverse reactions, the mechanisms of bacterial resistance, the mechanisms of action of antibiotics, best practices during antibiotic prescription, and the treatment and diagnosis recommendations generated in the CPGs) [3,6,7,8]. Finally, designing, implementing, and strengthening the antimicrobial stewardship programs (ASPs) in healthcare institutions is recommended, given that they are adjusted according to international and national standards and consider each institution’s characteristics (e.g., the level of complexity) [3,5,6,7].

Currently, the challenge for many countries, especially low- and middle-income countries (LMICs), lies in adequately designing and successfully implementing and operating these strategies [9,10]. This challenge worsened during the COVID-19 pandemic, which was a situation that may have unfortunately impacted antimicrobial overuse and increased AMR [11,12,13]. Colombia, a middle-income country in South America, also faces several challenges in adequately implementing these ASPs, such as a significant proportion of inhabitants in rural areas [14], an extended geographic and demographic diversity, and one of the highest inequality levels in Latin America (Gini coefficient in 2020 of 54.2) [15]. Although ASP studies have been conducted in some high-complexity institutions in Colombia [16], to date, there does not appear to be any published studies on the different strategies for improving antibiotic prescriptions in hospitals with different levels of complexity in Colombia.

This study aims to provide data on different strategies performed to improve antibiotic prescription in hospitals with different levels of complexity in Colombia, including developing and implementing CPGs, a continuing education course, a mobile consultation tool, and telemedicine. These are strategies that can be replicated or enhanced in other health institutions in Colombia as well as in other countries in the region.

## 2. Results

The interventions were carried out during the present study (2020–2022) to improve antibiotic prescription in three hospitals in Colombia with different levels of complexity, and the results of the indicators measured in the implemented ASP framework are discussed in the following sections.

### 2.1. Clinical Practice Guidelines

New CPGs were developed in the Colombian context to be used for the most common antibiotic use scenarios in the hospital environment: community-acquired pneumonia (CAP) [17] and surgical antimicrobial prophylaxis (SAP) [18].

In 2020, locally developed CPGs for the diagnosis and antimicrobial treatment of uncomplicated UTI [19] and skin and soft tissue infections (SSTIs) [20] were available, and their recommendations and good practice points were considered adequate and in force. In addition, the CPG for the diagnosis and management of intra-abdominal infections (IAIs) was in an advanced stage of development [21]. The development of all the CPGs involved the participation of clinical experts in relevant areas according to the topic. The recommendation development process was achieved by consensus using the Delphi technique according to the best available evidence and considering the local context.

### 2.2. Continuing Education and Quick Consultation

For disseminating and implementing the recommendations generated in the CPGs and strengthening knowledge about ASPs, the following strategies were designed and developed: (I) a Massive Open Online Course (MOOC) “Prudent use of antimicrobials in the hospital setting” (“Uso prudente de antimicrobianos en el entorno hospitalario”, title in the original language) launched on the Coursera platform in August 2021 [22]; and (II) a mobile application (app) “PROA UNAL” published in the AppStore and Play Store in October 2021 [23,24]. Both strategies are in the Spanish language and are aimed at healthcare personnel involved in the processes of prescription, administration, and control of antimicrobials in the hospital environment, including professionals, subspecialty students, and undergraduates.

The MOOC was designed as a continuing education strategy with flexibility in terms of time and location for compliance and learning. The course included recommendations for the appropriate diagnosis and use of antibiotics for CAP, UTIs, IAIs, SSTIs, and SAP; complementary readings; and formative assessments using a real-world case-based approach. To provide a complete approach to the topic, the course also included separate modules for the following topics: (I) the principles of pharmacology and antimicrobial resistance, (II) the main factors to account for when prescribing antimicrobials, (III) the main features of the ASPs, and (IV) main infection prevention and control strategies in the hospital environment. The app was designed as an easily and quickly accessible consultation tool for use in clinical practice, and includes timely recommendations and best practice points for diagnosing and using antibiotics for CAP, UTIs, IAIs, SSTIs, and SAP. From the launch date to August 2022, a total of 1861 unique learners enrolled in the MOOC, and a total of 620 unique users downloaded the app.

### 2.3. Antimicrobial Stewardship Programs’ Design and Implementation

According to the health institution’s level of complexity, an ASP was designed following the guidelines developed by the Ministry of Health of Colombia in collaboration with the Colombian Association of Infectious Diseases (ACIN) [25].

In Hospital Universitario Nacional de Colombia (HUN), a high-complexity hospital located in the capital city, the ASP consisted of an infectious disease specialist, nursing professional and assistant, microbiology professional, pharmaceutical chemist, an epidemiologist with experience in ASPs, representatives of different clinical and surgical specialties, and an administrative representative. In Hospital San Antonio de Soatá (HSAS), located 248 km from Bogotá in the vicinity of the Magdalena River, and in Hospital Jose Cayetano Vasquez (HJCV), located 240 km from Bogotá in the Andean mountains, including the infectious disease specialist and the epidemiologist with experience in ASP was made possible by using a telemedicine system and having a general physician of liaison in each hospital who received ASP training participate.

Once the ASP was designed and formed, with the support of the administrative team of each hospital, the program was institutionalized and made official between September and November 2021. The ASP was disclosed to the health professionals explaining the global problem of bacterial resistance, the importance of proper antibiotic use, the ASP’s role, and its mechanism of action that would be implemented in daily clinical practice in the hospital through active surveillance and post-prescription review with feedback (PPRF) [5,6,7]. Academic meetings were carried out with the physicians of different clinical and surgical specialties to present, discuss, and reinforce the recommendations for antibiotic use proposed in the CPGs, and to encourage continuous learning.

### 2.4. Indicators Measured in the ASP Framework

The final phase of the ASP implementation process in each hospital consisted of establishing an indicator measurement that would allow for knowing the program’s scope and progress, and whose analysis would allow for identifying opportunities to improve the ASP and establish new objectives. Accounting for the quality of the available information sources, the two indicators initially implemented were (I) adherence to the recommendations proposed in the CPGs, and (II) antibiotic use measured by the defined daily dose (DDD) per 100 occupied bed days.

#### 2.4.1. Adherence to the Recommendations Proposed in the CPGs

Adherence to the antibiotic recommendations proposed in the CPGs for CAP, uncomplicated UTIs, IAIs, SSTIs, and SAP was obtained using the following formula:

(1) $$\frac{a\;\times \;100}{b}$$

where *a* is the number of adult inpatients with antibiotic selection for the condition according to the guideline, and *b* is the number of adult inpatients with the condition. The specific conditions selected for measuring this indicator in the three hospitals are summarized in Table 1.

Measuring the adherence to the antibiotic recommendations proposed in the CPGs for CAP, uncomplicated UTIs, IAIs, and SSTIs was initially implemented only in the general ward or intensive care unit (ICU) for adult inpatients. Measuring the adherence to the antibiotic recommendations proposed in the CPG for SAP included elective, emergency, outpatient, or inpatient surgical operations in adult patients.

During the ASP intervention period in 2022, adherence to the recommendations proposed in the CPGs was assessed in a total of 1648 cases with antibiotics prescribed for the selected conditions (854 HUN, 298 HJCV, and 496 HSAS). Table 2 shows the total number of cases of antibiotic prescriptions assessed by hospital for each CPG. In all three institutions, a low number of CAP and mainly uncomplicated UTI cases with months of only two, one, or zero prescriptions were identified.

**Figure 1:**
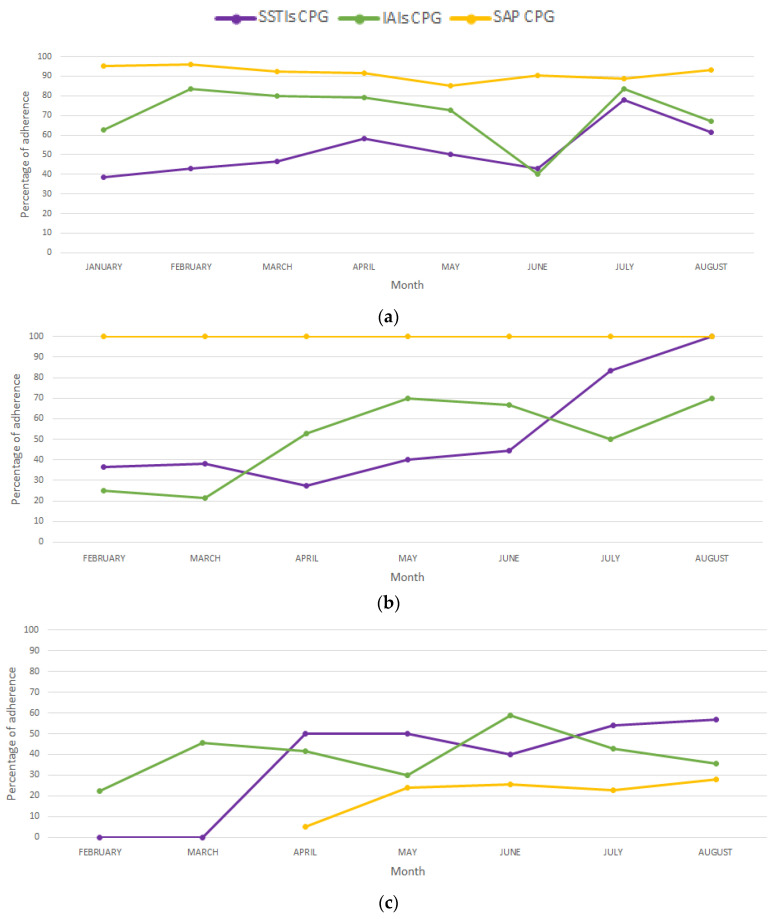
Trends in adherence to the antibiotic recommendations proposed in the CPGs for skin and soft tissue infections (SSTIs), intra-abdominal infections (IAIs), and surgical antimicrobial prophylaxis (SAP) during the antimicrobial stewardship program’s operation in 2022. (**a**) Hospital Universitario Nacional de Colombia (HUN). (**b**) Hospital Jose Cayetano Vasquez (HJCV). (**c**) Hospital San Antonio de Soatá (HSAS).

Table 3 presents the percentages of adherence to the antibiotic recommendations proposed by hospital for each CPG during each month of the ASP’s operation in 2022. Considering the large number of months with zero or one prescription identified for uncomplicated UTIs, these data cannot be adequately interpreted, so they are not shown. The temporal evolution of adherence to the antibiotic recommendations proposed in the CPGs for SSTIs, IAIs, and SAP, is shown in Figure 1. Adherence to the antibiotic recommendations for managing SSTIs increased progressively in the three hospitals. For IAIs, there was also an improvement in adherence in all three institutions, with the greatest increase in HJCV, which changed from 25% (3/12) in February to 70% (7/10) in August. Regarding adherence to the antibiotics recommended in the CPG for SAP, it was not possible to observe any changes in the HJCV since adherence was 100% throughout the study period. In the HUN, although the adherence was always ≥ 85%, there was a slight decrease in adherence over time. The HSAS showed the greatest increase, changing from 5% (2/39) in April to 28% (14/50) in August.

#### 2.4.2. Antibiotic Use Measured in DDD

The DDD per 100 occupied bed days calculated according to the Anatomical Therapeutic Chemical Classification and Defined Daily Dose System (ATC/DDD) instituted by the WHO [26] was obtained using the following formula:

(2) $$\frac{a}{b}\;\times \;\frac{100}{n\;\times \;Oc\;\times \;t}$$

where a is the consumption of the antibiotic during the analyzed period in grams, b is the WHO-recommended DDD for the analyzed antibiotic, n is the number of beds, Oc is the percentage of the occupation, and t is the time analyzed in days.

The DDD indicator was measured in the adult general ward and ICU. The indicator was initially implemented for ceftriaxone, piperacillin–tazobactam, ciprofloxacin, and meropenem, considering (I) that penicillins, cephalosporins, and fluoroquinolones have been reported as some of the most prescribed antibiotics in Colombian patients [27,28]; (II) the progressive increase in resistance to piperacillin–tazobactam, ciprofloxacin, and carbapenems reported in Colombia [29,30,31,32]; (III) that, due to their relatively high risk of bacterial resistance selection, these antibiotics were considered to be prioritized as key ASP targets and for monitoring, and were included in the Watch group of the WHO AWaRe classification of antibiotics [33]; (IV) that, due to the increasing resistance of *Escherichia coli*, *Klebsiella* spp., *Enterobacter* spp., *Morganella* spp., *Proteus* spp., and *Serratia* spp. to third-generation cephalosporins and carbapenems, they are included in the prioritization for developing new antibiotics by the WHO [34].

Figures S1 and S2 in the Supplementary Materials show the comparison of DDDs in the general ward and ICU, respectively, prior to ASP implementation in 2021 and with ASP implementation in 2022, for each antibiotic by the hospital. In the general ward, overall, there was a lower use of all four antibiotics in the three hospitals with the ASP, with the greatest decrease in ceftriaxone use at HSAS. The only two exceptions with an increase in antibiotic use were ceftriaxone consumption at HUN and meropenem consumption at HSAS; however, both cases had a DDD < 4, which is considered low consumption. In the ICU, overall, there was a lower use of all four antibiotics with the ASP at two medium-complexity hospitals located in Boyacá, with the greatest decrease in ciprofloxacin use at HJCV. In the ICU, at a high-complexity hospital located in Bogotá, there was lower carbapenem use, with a slight increase in ceftriaxone and piperacillin–tazobactam use.

In the high-complexity hospital, the increase in ceftriaxone consumption observed in the general ward and ICU was explained by its use for complicated UTIs and infections of gastrointestinal origin (e.g., diarrhea or liver abscess), which are considered appropriate prescriptions. Additionally, the increased consumption of piperacillin–tazobactam in the ICU was considered an effect of the carbapenem-sparing strategy, which resulted in an effective decrease in meropenem consumption. The increase in meropenem consumption observed in the general ward at one of the medium-complexity hospitals was due to the low availability of ertapenem, which made it necessary to use another carbapenem option available when the susceptibility pattern of the microorganism so indicated.

## 3. Discussion

This before-and-after study, with a multifaceted intervention that included developing the CPGs, continuing education through MOOCs, using quick consultation tools such as mobile apps, and using telemedicine in hospitals of different complexities and geographic locations, shows that ASPs can be implemented with positive results in terms of adherence and lower antimicrobial consumption in an environment of limited resources. This is one of the first comprehensive studies undertaken in a Latin American country such as Colombia to provide data on different strategies performed to improve antibiotic prescription in hospitals with different levels of complexity.

Developing clinical practice guidelines for the most common scenarios involving antibiotic use, adapted specifically to the Colombian context [17,18,19,20,21], was pivotal to this intervention. This aligned with the findings reported in a review published in 2019 by Cabrera and Pardo, regarding guidelines using the GRADE methodology, which was developed in Latin America and the Caribbean over the last 10 years [35], in which 81% were developed within the last 4 years and 68% were from Colombia. Although only 10% corresponded to infectious or communicable diseases, which highlights the need to continue developing guidelines for infectious diseases in the region.

The availability of a new course and a mobile application, both in the Spanish language and developed in a Latin American country, with a total of 1,861 unique students enrolled in the course and 620 unique users, is a significant achievement for the country and region considering the following factors: (I) the findings of studies on knowledge, attitudes, and practices about antibiotic use carried out on graduated physicians and medical students in Colombia show the need to strengthen the training of health professionals, subspecialty, and undergraduate students on antibiotic use and bacterial resistance [36,37]; (II) the findings of the scoping review developed in the LMICs by Wilkinson et al. reveal that very few studies were conducted in the Americas region that included educational interventions, such as training sessions or courses, to reduce inappropriate antibiotic prescription, with none conducted in Colombia [38]; (III) the findings of the systematic review published in 2020 by Helou et al. show that only two studies conducted in Latin America included app use by physicians treating in-hospital patients [39], both carried out in Brazil.

Concerning ASP design and implementation according to the health institution’s level of complexity, we observed that, through the telemedicine system, with an adequate and clear action plan and the commitment of the personnel, successfully implementing an ASP in medium-complexity hospitals located in small rural cities is possible. A barrier that was identified in these institutions with fewer numbers of health professionals is that there may be a much more frequent staff turnover, which continually restarts the ASP learning curve, their role and mechanism of action, and training on the importance of proper antibiotic use, which may reduce the impact and achievement of the program objectives. A similar effect of continually resetting the learning curve could occur in teaching hospitals due to the quarterly or even monthly service changes that are part of the curricula of subspecialty and undergraduate students, but this effect can be alleviated by having professors accompany them in their daily clinical practice and putting general physicians in charge of prescriptions. These findings are consistent with some of the organizational determinants to be considered for successfully implementing ASPs as suggested by Fabre et al., such as organizational support, organizational structure, human resources, and health worker priorities [10]. Additionally, this is in accordance with the findings of studies performed in the rural areas of Australia, a highly developed country [40], and in geographically dispersed areas of Brazil, an upper-middle-income country [41], which used telehealth for implementing ASPs and showed positive results.

Positive results were observed for the indicators measured in the ASP framework, with a progressive increase that adhered to the antibiotic recommendations proposed in the CPGs for managing SSTIs, IAIs, and SAP in the three hospitals. This aligns with the findings reported in the Cochrane systematic review developed by Davey et al., which concluded with high certainty evidence from 29 randomized controlled trials (RCTs) that implementing interventions aimed at healthcare professionals improves antibiotic prescriptions’ effectiveness by increasing antibiotic prescription guideline compliance compared to usual care [42]. No RCTs were conducted in Colombia, which reinforces the need to continue generating evidence from the country and region.

The assessment of antibiotic consumption, measured in DDD, showed that, overall, in the three hospitals, there was a lower use of antibiotics with the ASP, in both the general ward and ICU. Of course, with certain antibiotics, in which a different behavior was identified in some of the institutions, this observation could be related to certain indications for use and a possible change in the microbiological profile after the COVID-19 pandemic. These results align with the findings of a study carried out at provincial hospitals in an upper-middle-income country, Thailand, which reported how locally developed CPG implementation was effective in reducing ATB consumption [43]. Likewise, these results are consistent with the findings of a study performed at a teaching hospital located in the capital city of a lower-middle-income country, the Philippines, which showed that implementing an antimicrobial stewardship program can positively influence the quality of antibiotic prescription practices for SAP [44]. Additionally, these results are in accordance with the findings of a study carried out at high-complexity hospitals in Colombia, which reported decreasing antibiotic consumption with ASP implementation [45].

The interventions carried out in the present study to improve antibiotic prescription in hospitals with different levels of complexity in Colombia, which include the CPGs, MOOC, and apps, are a great step forward in the country’s current efforts for promoting prudent antibiotic use. The results of the indicators measured in the ASP framework obtained in the three hospitals showed that, in medium-complexity hospitals located in rural cities, successfully developing ASPs is possible when they are well-planned, implemented, and supported by the organization.

Colombia and other Latin American countries must continue to advance in activities that reduce AMR. For daily clinical practice and future research, we suggest the design, implementation, and improvement of these interventions aimed at optimizing antibiotic use in health institutions with different levels of complexity throughout the national territory. Additionally, there should be a focus on increasing the sustainability of and improving the already implemented strategies. All these activities are supported by public policies, such as Resolution 2471 published in December 2022, which adopts the technical guidelines for the Antimicrobial Use Optimization Programs in Colombia.

We are aware of our study’s limitations. These include the measured indicators’ short observation times in the ASP framework, which limits the performance of the statistical analysis. A low number of cases were identified for some conditions such as CAP and uncomplicated UTIs. When measuring the indicator of adherence to CPGs in the ASP framework, only five scenarios, considered the most prevalent with antibiotic use in the hospital setting, were included. However, considering the clinical significance of the results and the current need to interpret the results in clinical practice and research beyond their statistical significance, we believe that our findings and suggestions are robust and provide future direction.

## 4. Materials and Methods

### 4.1. Study Design and Setting

This was a before-and-after study with a multifaceted intervention carried out to improve antibiotic use in hospital environments. The study was conducted between 2020 and 2022 in the following three hospitals: Hospital Universitario Nacional de Colombia (HUN), a high-complexity private teaching hospital with a total of 230 beds (including 47 intensive care unit [ICU] beds) located in Bogotá, the capital city; two medium-complexity public hospitals located in Boyacá: Hospital San Antonio de Soatá (HSAS) with 45 beds (including 11 ICU beds open due to the COVID-19 pandemic) located in the municipality of Soatá, and Hospital Jose Cayetano Vasquez (HJCV) with 43 beds (including 16 ICU beds open due to the COVID-19 pandemic) located in the municipality of Puerto Boyacá.

### 4.2. Outcomes

The primary outcome was implementing interventions designed and developed to improve antibiotic use in hospitals with different complexities: CPGs, continuing education courses, quick consultation tools, and ASPs using telemedicine. The secondary outcomes evaluated were the indicators measured in the ASP framework, which included adherence to the recommendations proposed in the CPGs, and antibiotic consumption in the general ward and ICU.

### 4.3. Data Collection and Analysis

Regarding the indicators measured within the ASP framework, to measure the indicator of adherence to the recommendations proposed in the CPGs, the patient data were collected from the electronic healthcare records of each hospital and, using REDCap, which is an application based on a website designed to support data capture, the data were recorded on an electronic case report form (e-CRF) that was pre-validated and developed specifically for this purpose [46]. For the antibiotic use indicator measured in DDD, the antibiotic use data were collected from the pharmaceutical service records at each hospital and entered into Microsoft Excel^®^ in a specific form that was pre-validated and developed for this purpose. No formal sample size calculations were performed. A descriptive analysis was performed using the statistical software Stata^®^ version 17.0 (Stata Corp., College Station, TX, USA), with frequencies and proportions for the qualitative variables and measures of the central tendency and dispersion for the quantitative variables.

### 4.4. Ethical Considerations

This study’s activities were carried out within the framework of the WHO and PAHO action plan on antibiotic resistance and by following the guidelines developed by the Ministry of Health of Colombia for designing and implementing ASPs in the hospital environment, which is the framework for integrating this study’s activities with the hospital care provided to patients, without any interventions or interactions involving patients outside of their usual hospital care. The information collected from the medical records of the patients for measuring the indicators in the ASP framework was kept confidential. The final database was de-identified by anonymizing the personal data and assigning a reference number to each patient for verification purposes. Permission for implementing the ASP was granted by the management of each hospital. In addition, this study was approved by the Research Ethics Committee of HUN “Registration No. CEI-HUN-ACTA-2020-03. ID CEI-2020-03-06”.

## 5. Conclusions

By appropriately designing and implementing multifaceted strategies that consider the needs and characteristics of the local context, including developing CPGs, continuing education courses, using quick consultation tools, and using telemedicine for developing ASPs, improving antibiotic prescription in hospitals with different levels of complexity and geographic locations in middle-income settings is possible.

## Figures and Tables

**Table 1:** Conditions selected for measuring the indicator of adherence to CPGs.

Clinical Practice Guideline	Condition
Community-acquired pneumonia	Moderate CAP Severe CAP
Uncomplicated urinary tract infections	Cystitis Pyelonephritis
Intra-abdominal infections	Appendicitis Cholecystitis
Skin and soft tissue infections	Cellulitis Abscess Necrotizing fasciitis
Surgical antimicrobial prophylaxis	Neurosurgery ^1^ Cardiac surgery ^1^ Urological surgery ^1^ Gynecological surgery Colorectal surgery ^1^ Orthopedic surgery ^2^

^1^ Evaluated only in HUN. ^2^ Not evaluated in HUN.

**Table 2:** Number of cases of antibiotic prescriptions assessed for the indicator of adherence to CPGs during the antimicrobial stewardship program’s operation in 2022.

	CAP			UTIs			SSTIs			IAIs			SAP		
	HUN	HJCV	HSAS	HUN	HJCV	HSAS	HUN	HJCV	HSAS	HUN	HJCV	HSAS	HUN	HJCV	HSAS
January	6	ND	ND	1	ND	ND	13	ND	ND	8	ND	ND	83	ND	ND
February	3	2	14	2	2	1	7	11	7	6	12	9	78	21	ND
March	4	0	4	2	1	1	15	8	1	10	14	11	91	22	ND
April	5	0	14	0	0	1	12	11	10	14	17	12	83	21	39
May	4	2	13	4	0	0	10	10	16	11	20	10	40	25	42
June	3	3	24	0	0	0	7	9	10	5	12	17	94	16	39
July	3	1	16	0	0	4	9	6	13	6	4	14	89	16	66
August	4	1	13	0	0	4	13	3	7	6	10	14	103	18	50

CAP: community-acquired pneumonia. UTIs: urinary tract infections. IAIs: intra-abdominal infections. SSTIs: skin and soft tissue infections. SAP: surgical antimicrobial prophylaxis. HUN: Hospital Universitario Nacional de Colombia. HJCV: Hospital Jose Cayetano Vasquez. HSAS: Hospital San Antonio de Soatá. ND: not data.

**Table 3:** Percentages of adherence to the antibiotic recommendations proposed in CPGs during the antimicrobial stewardship program’s operation in 2022.

	CAP			SSTIs			IAIs			SAP		
	HUN	HJCV	HSAS	HUN	HJCV	HSAS	HUN	HJCV	HSAS	HUN	HJCV	HSAS
January	83	ND	ND	38	ND	ND	63	ND	ND	95	ND	ND
February	67	0	14	43	36	0	83	25	22	96	100	ND
March	75	NA	0	47	38	0	80	21	45	92	100	ND
April	100	NA	29	58	27	50	79	53	42	92	100	5
May	75	50	77	50	40	50	73	70	30	85	100	24
June	67	67	58	43	44	40	40	67	59	90	100	26
July	67	100	44	78	83	54	83	50	43	89	100	23
August	75	100	46	62	100	57	67	70	36	93	100	28

CAP: community-acquired pneumonia. IAIs: intra-abdominal infections. SSTIs: skin and soft tissue infections. SAP: surgical antimicrobial prophylaxis. HUN: Hospital Universitario Nacional de Colombia. HJCV: Hospital Jose Cayetano Vasquez. HSAS: Hospital San Antonio de Soatá. NA: not applicable. ND: not data.

## Data Availability

Additional data are available upon reasonable request from the corresponding authors.

## Supplementary Materials

The following supporting information can be downloaded at: https://www.mdpi.com/article/10.3390/antibiotics12050867/s1, Figure S1: General ward antibiotic use measured in DDD per 100 occupied bed days for broad-spectrum antibiotics; Figure S2: Intensive care unit antibiotic use measured in DDD per 100 occupied bed days for broad-spectrum antibiotics.
