# Supplementary material for: Interventions to Improve Antibiotic Use in Hospitals with Different Levels of Complexity in Colombia: Findings from a Before-and-After Study and Suggestions for the Future
Source: Antibiotics (Basel). 2023 May 7;12(5):867. doi: 10.3390/antibiotics12050867 (PMC10215773; doi:10.3390/antibiotics12050867)
Supplement: Supplementary file 1 [file antibiotics-12-00867-s001.zip › antibiotics-2265143-supplementary.pdf]

Interventions to Improve Antibiotic Use in Hospitals with Different Levels of Complexity in Colombia: Findings from a Before-and-After Study and Suggestions for the Future

Supplementary Material

Figure S1. General ward antibiotic use measured in DDD per 100 occupied bed days for broad-spectrum antibiotics. Comparison 2021 prior to implementation of the antimicrobial stewardship program versus 2022 during the antimicrobial stewardship program's operation.

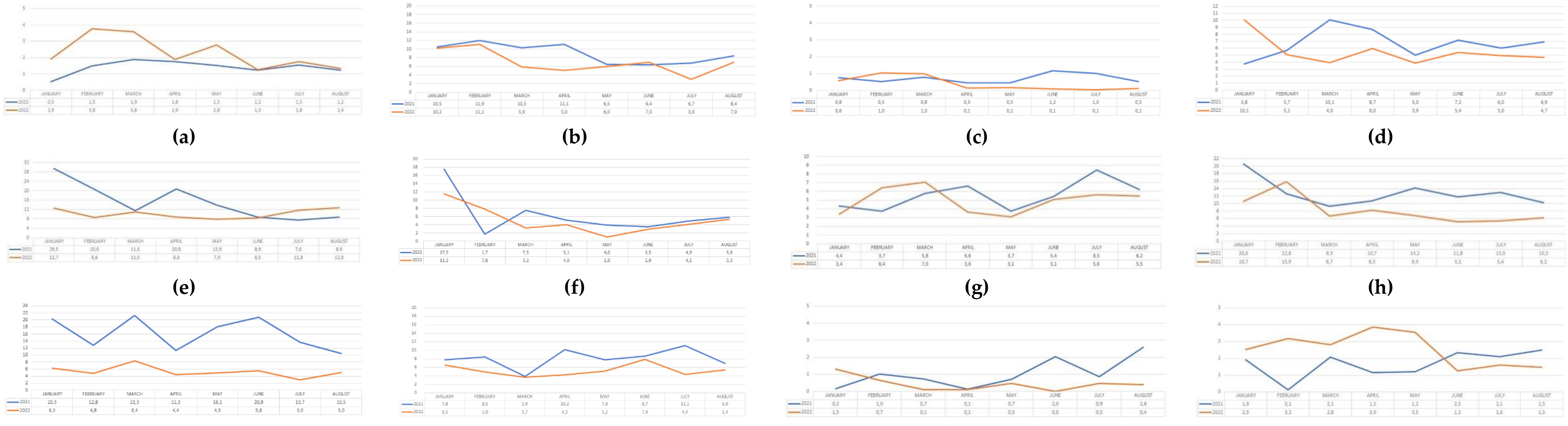

(a) **(b)** **(c)** **(d)**

(e) **(f)** **(g)** **(h)**

(i) **(j)** **(k)** **(l)**

(**a**) ceftriaxone HUN; (**b**) piperacillin tazobactam HUN; (**c**) parenteral ciprofloxacin HUN; (**d**) meropenem HUN; (**e**) ceftriaxone HJCV (**f**) piperacillin tazobactam HJCV; (**g**) parenteral ciprofloxacin HJCV; (**h**) meropenem HJCV; (**i**) ceftriaxone HSAS (**j**) piperacillin tazobactam HSAS; (**k**) parenteral ciprofloxacin HSAS; (**i**) meropenem HSAS.

Figure S2. Intensive care unit antibiotic use measured in DDD per 100 occupied bed-days for broad-spectrum antibiotics. Comparison 2021 prior to implementation of the antimicrobial stewardship program versus 2022 during the antimicrobial stewardship program's operation.

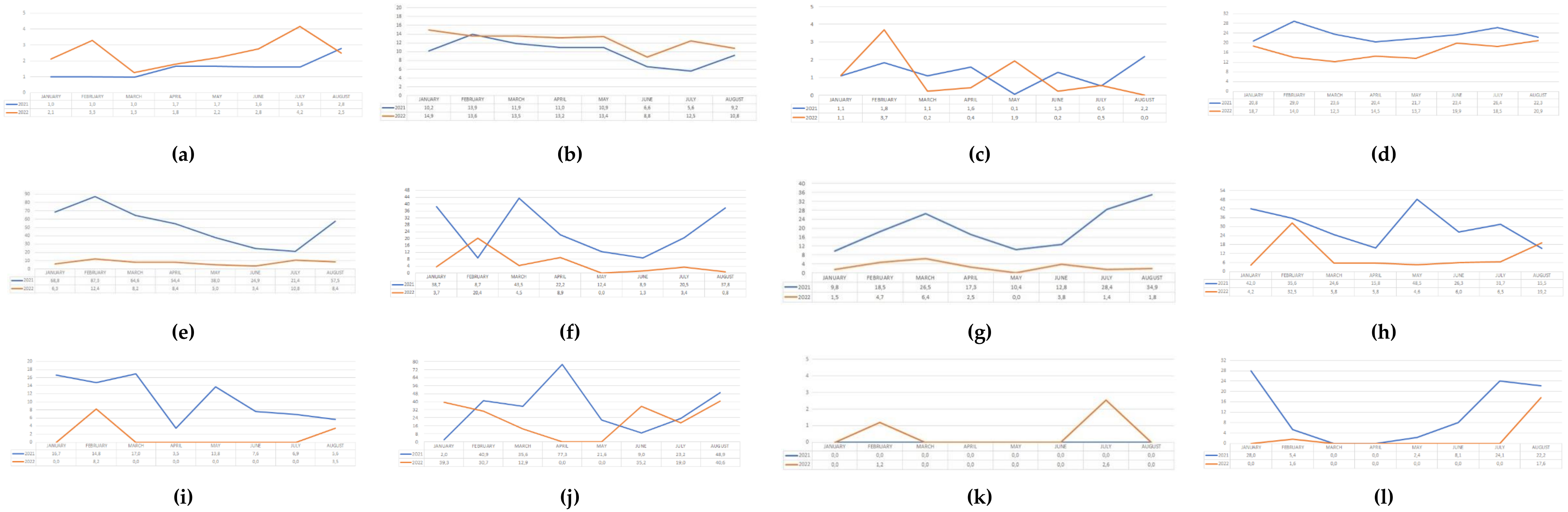

(a) ceftriaxone HUN; (b) piperacillin tazobactam HUN; (c) parenteral ciprofloxacin HUN; (d) meropenem HUN; (e) ceftriaxone HJCV (f) piperacillin tazobactam HJCV; (g) parenteral ciprofloxacin HJCV; (h) meropenem HJCV; (i) ceftriaxone HSAS (j) piperacillin tazobactam HSAS; (k) parenteral ciprofloxacin HSAS; (i) meropenem HSAS.
